# Supplementary material for: DAPK1‐Mediated Parkin Inactivation Enhances Neurotoxicity via MITOL‐Dependent Degradation
Source: J Cell Mol Med. 2026 Apr 6;30(7):e71132. doi: 10.1111/jcmm.71132 (PMC13053296; doi:10.1111/jcmm.71132)
Supplement: Supplementary file 1 — Figure S1: Co‐immunoprecipitation reveals interaction between parkin and DAPK1 in the absence of MG132. Where indicated, HEK293 cells were transfected for 24 h with plasmids encoding Myc‐parkin or FLAG‐DAPK1 alone or in combination. Cell lysates were subjected to immunoprecipitation using an anti‐FLAG antibody, and the resulting immunoprecipitates were immunoblotted with the indicated antibodies. Figure S2: DAPK1 decreases the protein stability of parkin. (A) MN9D cells were transfected for 24 h with plasmids encoding Myc‐parkin alone or together with increasing amounts of FLAG‐DAPK1. Cell lysates were immunoblotted with the indicated antibodies. Relative levels of parkin were quantified and the results presented as the mean ± SD of three independent experiments (***p ≤ 0.0001; **p ≤ 0.001). (B) MN9D cells were transfected for 24 h with plasmids encoding Myc‐parkin alone or in combination with FLAG‐DAPK1‐WT or FLAG‐DAPK1‐KD. Cell lysates were immunoblotted with the indicated antibodies. Relative levels of parkin were quantified and results presented as the mean ± SD of three independent experiments (***p ≤ 0.0001). (C) The mouse embryo fibroblasts (MEF) expressing wild‐type DAPK1 (DAPK1‐WT) or its knockout (DAPK1‐KO) MEFs were mock‐transfected or transfected for 24 h with FLAG DAPK1. Cell lysates were immunoblotted with the indicated antibodies. Relative levels of parkin were quantified and the results presented as the mean ± SD of three independent experiments (***p ≤ 0.0001; **p ≤ 0.001). (D) MN9D cells were transfected for 24 h with plasmids encoding Myc‐parkin alone or together with FLAG‐DAPK1. Cells were treated for the indicated times with 25 μg/mL cycloheximide (CHX), and cell lysates were immunoblotted with the indicated antibodies. Relative levels of parkin were quantified and the results presented as the mean ± SD of three independent experiments (*p ≤ 0.05). (E) Where indicated, MN9D cells were transfected for 24 h with plasmids encoding Myc‐parkin, along [file JCMM-30-e71132-s001.docx]

**Supplementary Figures**


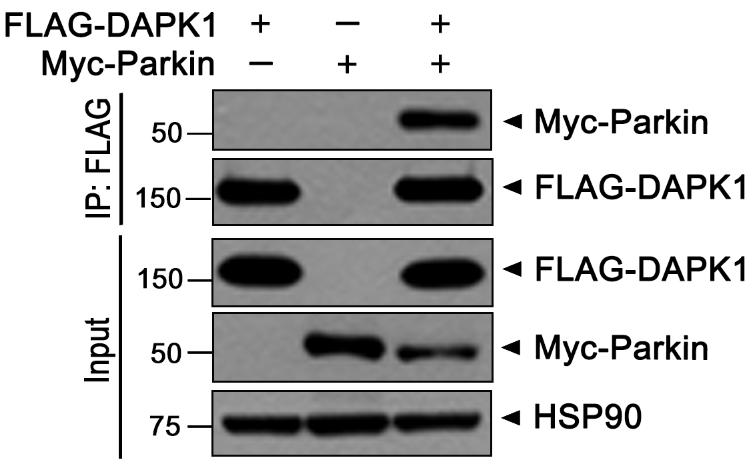


**Fig. S1** **Co-immunoprecipitation reveals interaction between parkin and DAPK1 in the absence of MG132.** Where indicated, HEK293 cells were transfected for 24 h with plasmids encoding Myc-parkin or FLAG-DAPK1 alone or in combination. Cell lysates were subjected to immunoprecipitation using an anti-FLAG antibody, and the resulting immunoprecipitates were immunoblotted with the indicated antibodies.


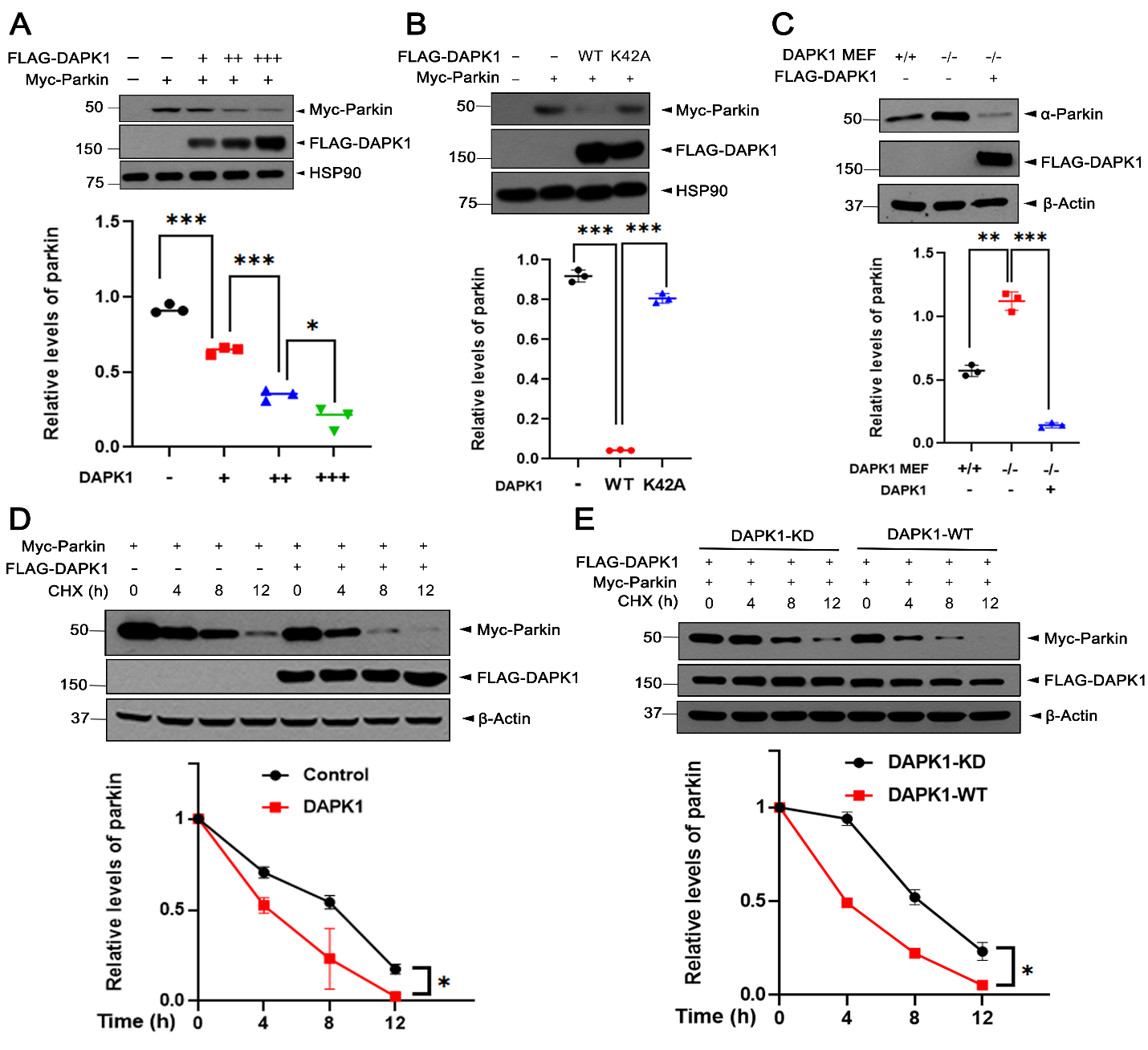


**Fig. S2 DAPK1 decreases the protein stability of parkin.** (A) MN9D cells were transfected for 24 h with plasmids encoding Myc-parkin alone or together with increasing amounts of FLAG-DAPK1. Cell lysates were immunoblotted with the indicated antibodies. Relative levels of parkin were quantified and the results presented as the mean ± SD of three independent experiments (****p* ≤ 0.0001; ***p* ≤ 0.001). (B) MN9D cells were transfected for 24 h with plasmids encoding Myc-parkin alone or in combination with FLAG-DAPK1-WT or FLAG-DAPK1-KD. Cell lysates were immunoblotted with the indicated antibodies. Relative levels of parkin were quantified and results presented as the mean ± SD of three independent experiments (****p* ≤ 0.0001). (C) The mouse embryo fibroblasts (MEF) expressing wild-type DAPK1 (DAPK1-WT) or its knockout (*DAPK1*-KO) MEFs were mock-transfected or transfected for 24 h with FLAG DAPK1. Cell lysates were immunoblotted with the indicated antibodies. Relative levels of parkin were quantified and the results presented as the mean ± SD of three independent experiments (****p* ≤ 0.0001; ***p* ≤ 0.001). (D) MN9D cells were transfected for 24 h with plasmids encoding Myc-parkin alone or together with FLAG-DAPK1. Cells were treated for the indicated times with 25 μg/ml cycloheximide (CHX), and cell lysates were immunoblotted with the indicated antibodies. Relative levels of parkin were quantified and the results presented as the mean ± SD of three independent experiments (**p* ≤ 0.05). (E) Where indicated, MN9D cells were transfected for 24 h with plasmids encoding Myc-parkin, along with either FLAG-DAPK1-KD or FLAG-DAPK1-WT. Cells were treated for the indicated times with 25 μg/ml CHX, and cell lysates were immunoblotted with the indicated antibodies. Relative levels of parkin were quantified and the results presented as the mean ± SD of three independent experiments (**p* ≤ 0.05). β-Actin and Hsp90 served as a loading control.


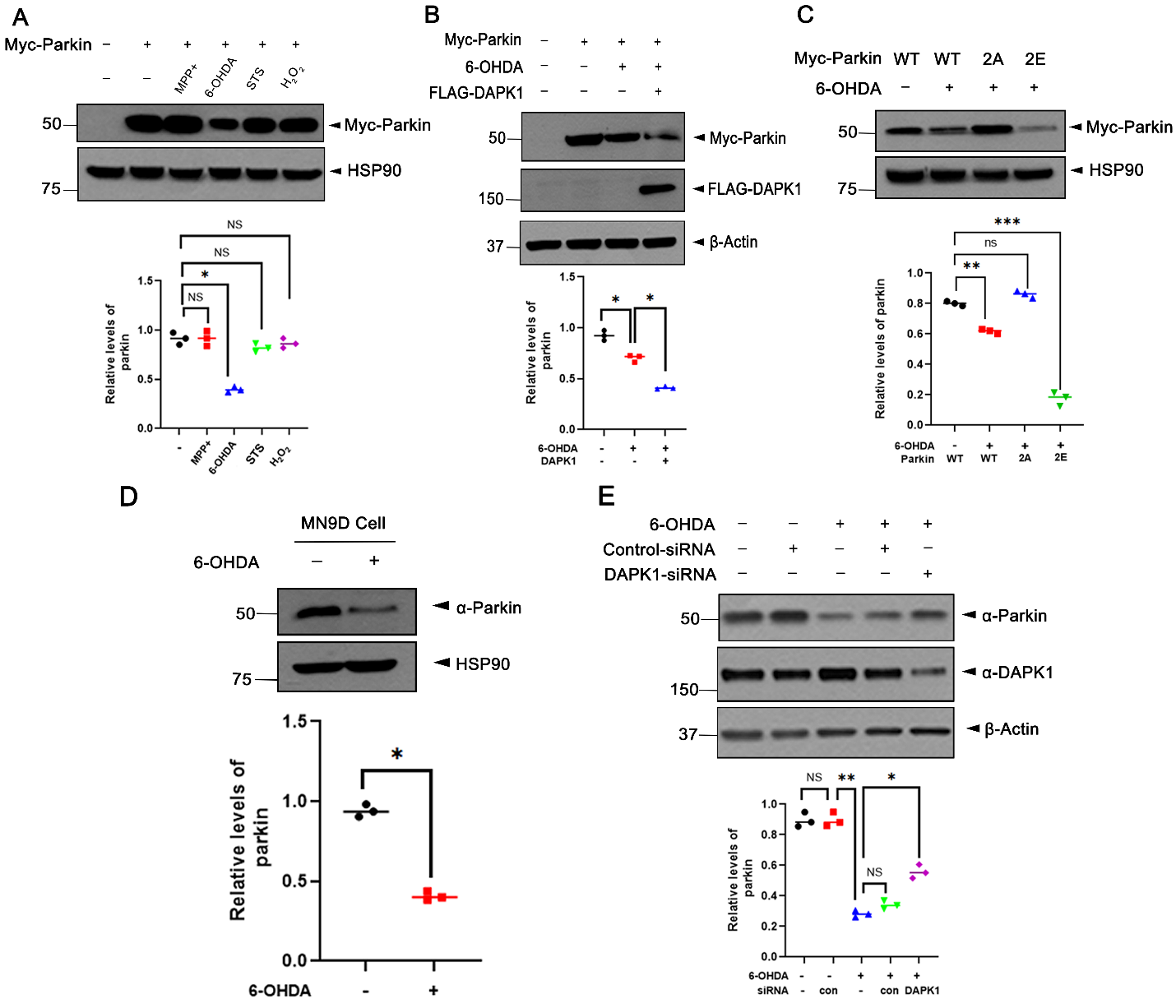


**Fig. S3 Exposure of MN9D cells to 6-OHDA caused the reduction of parkin.** (A) Where indicated, MN9D cells were treated for 6 h with MPP^+^ (500 μM), 6-OHDA (50 μM), staurosporine (STS, 0.5 μM), or H_2_O_2_ (500 μM). Cell lysates were immunoblotted with the indicated antibodies. Relative parkin levels were quantified and are presented as the mean ± SD of three independent experiments (**p* ≤ 0.05; NS, not significant). (B) MN9D cells were transfected for 24 h with plasmids encoding FLAG-DAPK1 alone or in combination with Myc-parkin, and treated for additional 6 h with 50 μM 6-OHDA. Cell lysates were immunoblotted with the indicated antibodies. Relative parkin levels were quantified and are presented as the mean ± SD of three independent experiments (**p* ≤ 0.05). (C) MN9D cells were transfected for 24 h with Myc-parkin-WT, Myc-parkin-2A, or Myc-parkin-2E and treated for additional 6 h with 50 μM 6-OHDA. Cell lysates were immunoblotted with the indicated antibodies. Relative parkin levels were quantified and are presented as the mean ± SD of three independent experiments (****p* ≤ 0.0001; ***p* ≤ 0.001; NS, not significant). (D) MN9D cells were treated for 6 h with vehicle (-) or 50 μM 6-OHDA. Cell lysates were immunoblotted with the indicated antibodies. Relative parkin levels were quantified and are presented as the mean ± SD of three independent experiments (**p* ≤ 0.05). (E) MN9D cells were transfected for 48 h with control siRNA or *DAPK1*-siRNA. Cells were then left untreated or treated for additional 4 h with 50 μM 6-OHDA, and cell lysates were immunoblotted with the indicated antibodies. Relative parkin levels were quantified and are presented as the mean ± SD of three independent experiments (***p* ≤ 0.001; **p* ≤ 0.05; NS, not significant). β-Actin and Hsp90 served as a loading control.


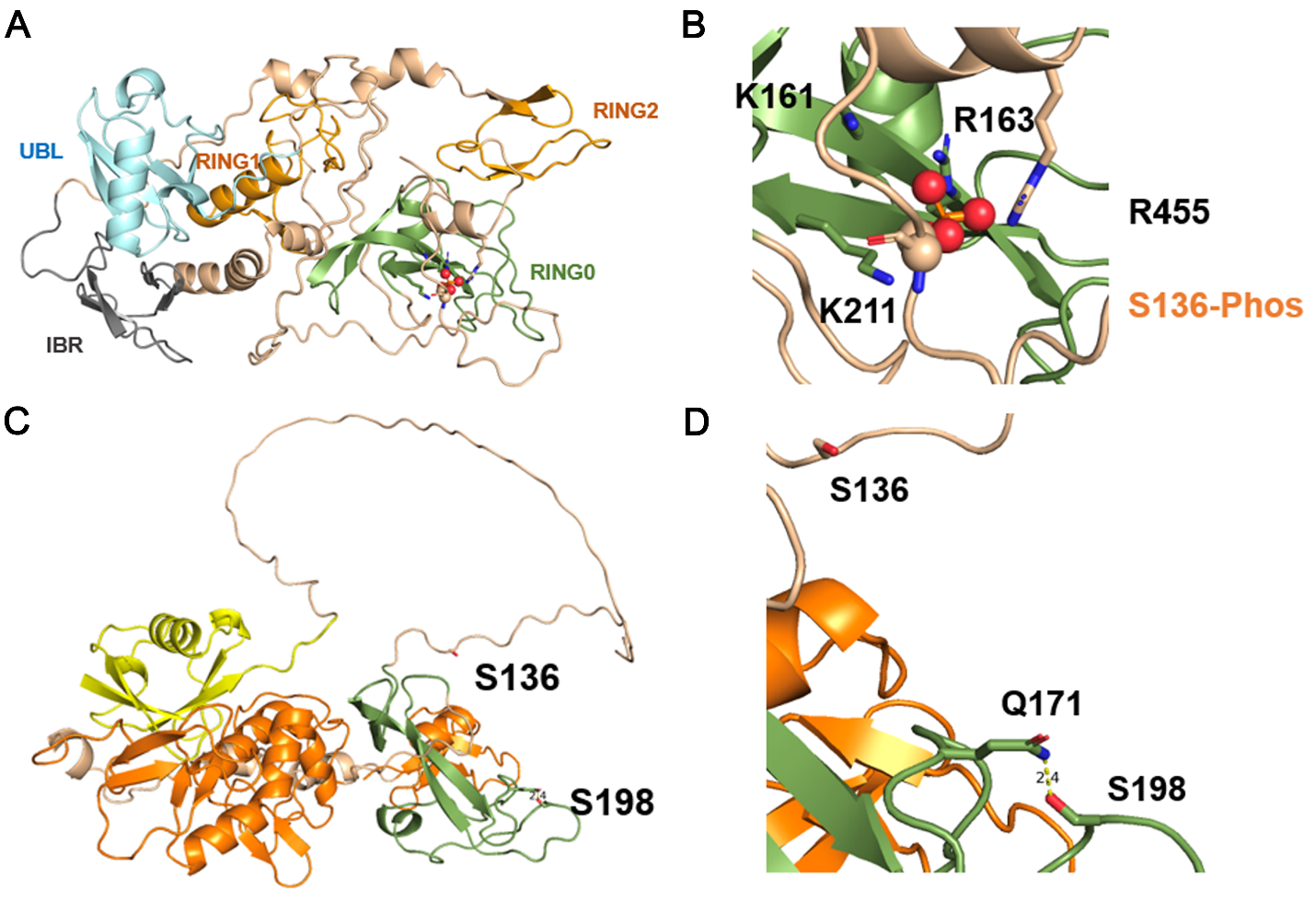


**Fig. S4 Predicted structure of phosphorylated Parkin.** (A, B) Ser136 and Ser198 of Parkin are highlighted in the predicted Parkin structure. Ser136 is located on the loop between UBL and RING0, while Ser198 forms a hydrogen bond with Q171. (C) Representative model of the AlphaFold3-predicted complex structure of Ser136-phosphorylated Parkin. (D) Basic amino acids interacting with phosphoserine 136 are highlighted.
